# The Prediction Model for Triple-Negative Breast Cancer Prognosis and Immunotherapy Efficacy Based on Single-Cell Sequencing of CD8+ T cells

**DOI:** 10.7150/jca.115507

**Published:** 2025-07-24

**Authors:** Jiarong Yi, Yejun Qiao, Zhengchong Xiong, Jikun Feng, Xiazi Zouxu, Shuang Zeyu, Xi Wang

**Affiliations:** 1 Department of Breast Oncology, Sun Yat-sen University Cancer Center, the State Key Laboratory of Oncology in South China, Collaborative Innovation Center for Cancer Medicine, Guangzhou, Guangdong, China.; 2 Fudan University Shanghai Cancer Center and Institutes of Biomedical Sciences, Shanghai Medical College, Fudan University, Shanghai, China.

**Keywords:** T cell single cell sequencing, immunotherapy, prognostic model

## Abstract

**Background:** Triple-negative breast cancer (TNBC) exhibits a higher propensity for recurrence, distant metastasis, and mortality than the other subtypes of breast cancer. TNBC is primarily attributed to the lack of expression of the estrogen receptor (ER), progesterone receptor (PR), and human epidermal growth factor receptor 2 (HER2).

**Methods:** Single-cell sequencing results of CD8^+^ T cells in TNBC patients were screened for differentially expressed and immune-related genes. The selected genes were then analyzed with immunohistochemistry for their prognostic effects. Additionally, a regression model was constructed to ascertain the gene expression score and classify patients into high- and low-risk groups. We further analyzed the impact of gene expression on prognosis based on risk grouping and evaluated its potential as a prognostic predictor for TNBC patients. This analysis was validated using PCR and the prognostic data from patient samples. We also investigated the effect of risk grouping on immunotherapy in TNBC patients and evaluated its potential to predict the efficacy of immunotherapy in TNBC patients.

**Results:** Single-cell sequencing of CD8^+^ T cells from TNBC patients identified 191 differentially expressed genes. Among them, XCL1, RASGRP1, CTSD, and AIP were reported to be independent prognostic factors for TNBC. The results were verified through immunohistochemistry. Additionally, a regression analysis model was constructed using these four genes to classify patients into risk groups. The high-risk group correlated with a poor prognosis in patients and could serve as an independent prognostic factor for TNBC. The results were further validated through PCR. Notably, patients in the low-risk group displayed a better response to immunotherapy.

**Conclusion:** Based on the single-cell sequencing results of CD8^+^ T cells from TNBC patients, a prediction model was established, which facilitated prognosis prediction in TNBC patients and evaluated the patients' response to immunotherapy. In summation, this model could potentially assist in improving the efficacy of TNBC immunotherapy.

## Background

Triple-negative breast cancer (TNBC) refers to a specific subtype of breast cancer that lacks expression of the estrogen receptor (ER), progesterone receptor (PR), and human epidermal growth factor receptor 2 (HER2) 1. TNBC accounts for approximately 15% of breast cancer cases and is characterized by its resistance to hormone therapy and HER2-targeted therapy 2. TNBC has reported higher incidences of recurrence, distant metastasis, and mortality than the other breast cancer subtypes, which correlates to the poor prognosis of TNBC patients 3. In patients without metastatic TNBC, the tumors exhibited a favorable response to neoadjuvant therapy, with approximately 40%-50% of tumors achieving a pathological complete response (pCR) 4. Tumors with pCR are normally associated with a lower 10-year recurrence rate in patients (i.e., less than 15%) as compared to patients with residual disease (i.e., more than 50%). Nonetheless, chemotherapy has been the primary first-line treatment option for TNBC patients, with a median overall survival (OS) of two to three years 5. Recently, targeted drugs and immunotherapies have reported significant progress in the prognosis of TNBC patients with high therapeutic potential. Nevertheless, only a few targeted drugs and immunotherapies are currently available for the clinical treatment of TNBC, such as the antibody-drug conjugate goxatuzumab and the anti-PD-1 agent pembrolizumab. However, immunotherapy has reported a low single-agent efficacy and a relatively high rate of drug resistance, which prompts the search for better alternative treatment strategies 6-9.

The CD8^+^ T cells can respond to tumor-specific antigens and autoantigens, and these cells can selectively target and kill cancer cells 10. However, CD8^+^ T cells are ineffective in tumor tissues, which suggests that the tumor-reactive CD8^+^ T cells are dysfunctional during tumorigenesis 11. Changes in the tissue microenvironment during tumorigenesis may have affected CD8^+^ T cell differentiation, resulting in a non-reactive T cell state. When primitive antigen-specific CD8^+^ T cells encounter antigens in the context of acute inflammation, the T cells undergo clonal expansion and differentiate into cytolytic effector T cells. After the pathogen or antigen is cleared, most effector T cells die, but a small number survive and form memory T cells 12, 13. The differentiation of T cells into their effector and memory states involves different transcriptional and epigenetic factors. Surface suppressor molecules of CD8^+^ T cells inhibit the tumor suppressor ability of CD8^+^ T cells and promote tumor escape by binding to tumor or regulatory immune cell surface ligands. Immunotherapies targeting cytotoxic T lymphocyte antigen 4 (CTLA-4) and programmed cell death 1 (PD-1) have been used for the treatment of melanoma, non-small cell lung cancer (NSCLC), renal cell carcinoma (RCC), and Hodgkin's lymphoma 14-21. However, immunotherapy resistance and poor patient response remain the key challenge for cancer treatment. Herein, this study analyzed the gene expression of CD8^+^ T cells and the expression of various signaling pathways. This study also evaluated the effect of CD8^+^ T cells on immunotherapy using an established prognostic model.

## Materials and Methods

### Gene expression dataset

The TNBC dataset was retrieved from the Cancer Genome Atlas (TCGA) and Gene Expression Omnibus (GEO) databases. This study only recruited patients diagnosed with TNBC with confirmed pathological and clinical information. Patients with insufficient or missing data, including age, TNM staging, and OS, were excluded. Information from 116 patients was retrieved. The GSE47994 dataset was retrieved from GEO database by searching the keywords "TNBC" and “survival", which contained information from 133 patients. Patient characteristics in TCGA and GSE47994 are displayed in Table 1. Subsequently, qRT-PCR and immunohistochemistry were performed on 40 samples collected from TNBC patients at the Sun Yat-sen University Cancer Center. The basic information of patients is displayed in Table 2.

### Differential expression analysis of CD8^+^ T cells based on single-cell sequencing

Based on the single-cell sequencing data in the TISCH database (Tumor Immune Single Cell Hub, http://tisch.comp-genomics.org), we screened the T-cell sequencing dataset of TNBC and selected CD8^+^ T cells from the GSE148673 dataset as the subject of our research. This dataset consisted of 46501 single cells from 21 tumors, including TNBC, pancreatic ductal adenocarcinoma, thyroid undifferentiated carcinoma, invasive ductal carcinoma, and glioblastoma. We selected 10359 cells from six TNBC patients and analyzed the immune cell types and composition to sequence CD8^+^ T cells (tools provided by TISCH).

### Differential gene expression analysis of T cells and associated immune genes

Sequencing results were used to compare genes in different states, and an intersection was made with immune-related genes (*R package of VennDiagram*). The intersection was then analyzed in *STRING* (https://cn.string-db.org) to assess their correlation, and genes with a correlation point ≥ 20 were considered hot genes.

### Single-factor analysis of hot-spot genes

A single-factor analysis was performed to screen genes that could serve as independent prognostic factors for the construction of models. TCGA data were used to conduct an independent prognostic analysis of hot-spot genes to determine their prognostic impact on TNBC patients.

### Identification of key prognostic genes with immunohistochemistry

Immunohistochemistry was performed on 40 samples collected from TNBC patients, and stain intensity was analyzed with recognition software. Based on the degree of staining, the samples were divided into either high expression group or low expression groups, using the imagine gray scale. And then prognostic analysis was conducted to determine the impact of these genes as independent prognostic factors on patient prognosis and survival.

### Immunohistochemistry

The tissues were first dewaxed in xylene, rehydrated in alcohol, and blocked in endogenous peroxidase activity. The tissues were then incubated overnight at 4 ℃ with specific antibodies targeting AIP, RASGRP1, CTSD, and XCL1 (rabbit; 1:100, Abcam, Cambridge, UK). The samples were incubated at room temperature with secondary antibodies (ab97080, goat anti-rabbit, 1:2,000; ab97040, goat anti-mouse, 1:500, Abcam) for 10 min and subsequently, in 3-3'-diamino-benzidine for 1.5 min. After that, the samples were counter-stained with hematoxylin for 30 s and visualized under a microscope. Median gray level was set as CUTOFF. Based on the degree of staining from grayscale imaging, the samples were divided into either high- or low-expression groups. The result and clinical information were then subjected to survival analysis using the* R package of survival and survminer*.

### Model construction and verification

Lasso regression analysis was conducted on selected genes to determine the best coefficients and regression equations (*R package of glmnet and survival*) 22. The CUTOFF was selected based on TCGA and GEO data to divide the high- and low-risk groups. TCGA data and the GSE47994 dataset were used to verify the accuracy of the model for prognosis prediction. Results from q-RT PCR were used to calculate the patient risk score based on the regression coefficient. The median was taken as CUTOFF, and the patients were divided into high- and low-risk groups. Finally, survival analysis was conducted based on the different risk groups. There are limits in lasso regression, including large amounts of computation, overfitting problems when dealing with complex data and high sensitivity of parameter selection, but it is still the most suitable choice for the study.

### qRT-PCR analysis

Total RNA was extracted from cultured vascular endothelial cells and fibroblasts with Trizol (Invitrogen, Carlsbad, USA). For mRNA detection, cDNA was synthesized from 1 μg of total RNA using the Revert Aid First-Strand cDNA Synthesis Kit (Fermentas, Burlington, Canada). qRT-PCR was then analyzed using the SYBR Premix ExTaqTM II configuration and the ABI PRISM® 7900HT system. The relative standard curve method (2^-ΔΔCT^) used GAPDH as a reference to detect the relative mRNA expression. The PCR primers used in this study are as follows:

AIP-qF: AGGCAGTGCCACTTATCCAC

AIP-qR: ACCCAGGCTGTTCCTTCATC

RASGRP1-qF: GGCTCCGCGGAAACCTT

RASGRP1-qR: TTCGGAACTGGGTGATGTGG

CTSD-qF: CTGGACATCGCTTGCTGGAT

CTSD-qR: TGCCTCTCCACTTTGACACC

XCL1-qF: AGGACCTCAGCCATGAGACT

XCL1-qR: TCACTCCCTACACCTTCCACA

The expression median was taken as CUTOFF, and the patients were divided into high- and low-risk groups.

### Validation of risk score

Based on the risk score and prognostic data from TCGA, univariate and multivariate analyses were conducted to evaluate the significance of selected genes to predict the prognosis of TNBC patients (*R package of survival*). Additionally, ROC analysis was performed to evaluate the accuracy of predicting the prognosis of TNBC patients (*R package of survival, survminer, and timeROC*).

### Mechanism

Based on the risk score and grading, we conducted GO and KEGG analysis using GSEA to study the differentially expressed signaling pathways and the potential mechanism of key genes influencing the prognosis of patients (*R package of limma, org.Hs.eg.db, clusterProfiler, and enrichplot*). Additionally, a waterfall map was plotted to further analyze the specific expression of signal pathways in the high- and low-risk groups (*R package of maftools*).

### Correlation analysis between risk score and immune function

Based on the risk score and grading, immune checkpoints with significant differential expression were screened, and the infiltration of immune cells in different risk groups was analyzed with CIBERSORT. The differences in immune cell function were also evaluated (*R package of limma, GSVA, GSEABase, ggpubr, and reshape2*) 23.

### Effect analysis of immunotherapy

Using the jarrydmartinx/metabric2 dataset, the prognosis of TNBC patients who had undergone immunotherapy with different risk scores was analyzed to validate the effectiveness of the prognosis assessment model (*R package of stringi, caret, glmnet, and survminer*). The effect of immunotherapy in different patients was further analyzed to evaluate the predictive function of the model in TNBC patients (*R package of limma*) 24.

#### Statistical Analysis

The R software (version 4.0.3) was used for all statistical analyses. Univariate and multivariate Cox regression analyses were performed to evaluate the survival situation. Hazard ratio (HR) and 95% confidence interval (CI) were calculated to identify genes related to overall survival. Unless noted otherwise, *P* < 0.05 was considered statistically significant.

## Results

### CD8^+^ T cell single-cell sequencing analysis

**Figure 1:**
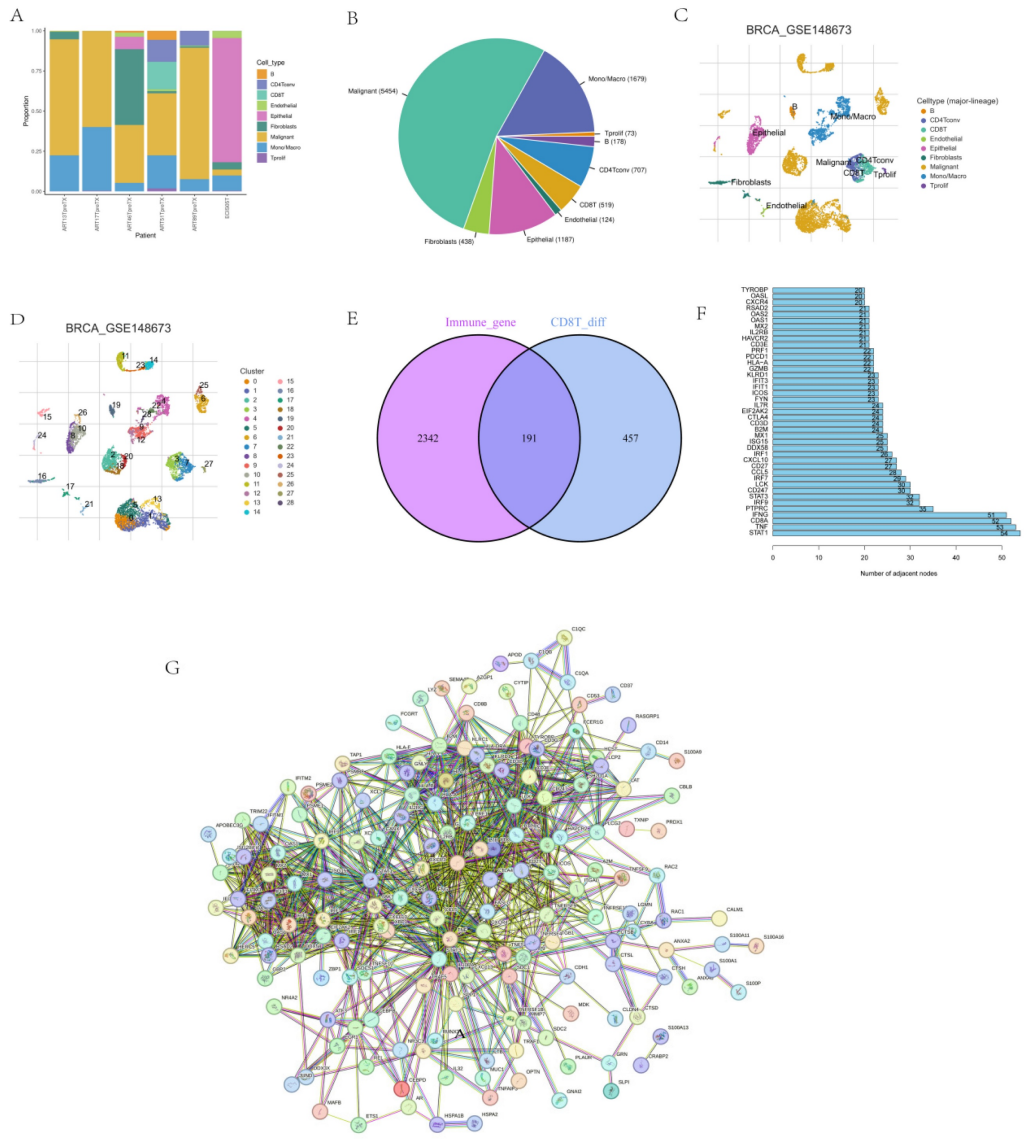
** CD8^+^ T cell single-cell sequencing analysis. (A-D)** Immune cell types and composition in CD8^+^ T cell single cell sequencing results. **(E-F)** Screening of hot genes, where G refers to the hot genes.

The immune cell types and composition were analyzed based on 10359 cells from six TNBC patients in the GSE148673 dataset (Figs. 1A-D), and the sequencing of CD8^+^ T cells was intersected with immune genes (Fig. 1E). STRING was used to further analyze intersection genes, and genes with association nodes greater than 20 were selected as hot genes (Figs. 1F-G).

### Model construction and verification

**Figure 2:**
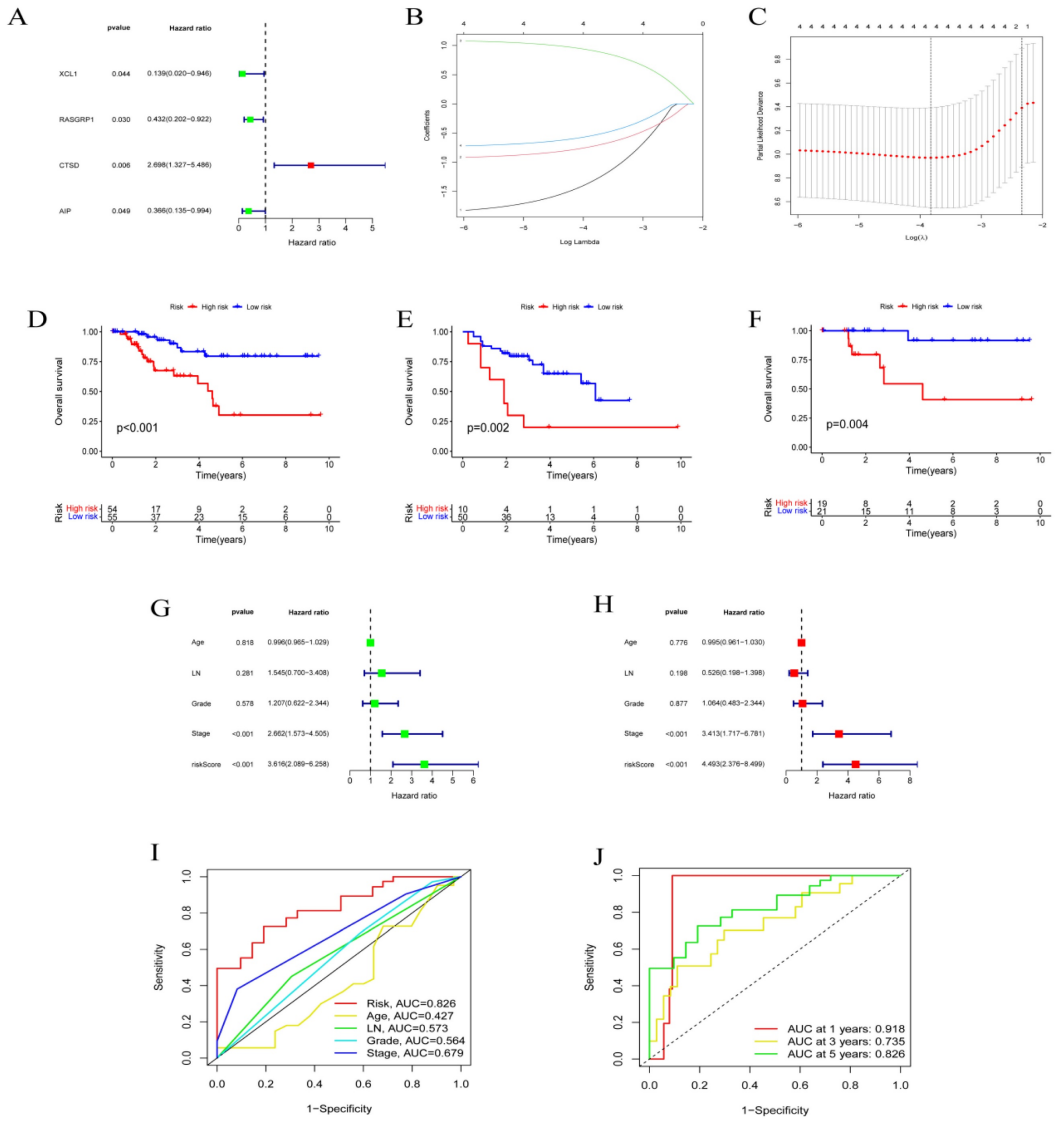
** Model construction and verification.** Results from **(A)** single factor analysis, **(B-C)** lasso analysis, **(D)** prognosis analysis based on TNBC, **(E)** prognosis analysis based on GEO, **(F)** prognosis analysis based on qRT-PCR, **(G)** univariate analysis, **(H)** multivariate analysis, and **(I-J)** ROC analysis.

Single-factor analysis was performed on each of the selected hot genes. Genes that could be used as independent prognostic factors were selected for the construction of the model, such as AIP, CTSD, RASGRP1, and XCL1 (Fig. 2A). The lasso regression model was constructed to determine the final coefficients of the genes. The XCL1 coefficient was -1.313, the RASGRP1 coefficient was -0.723, the CTSD coefficient was 0.909, and the AIP coefficient was -0.538 (Figs 2B-C). The patient risk score was calculated according to the regression coefficient, and the median was considered the CUTOFF. The prognoses of different risk groups in GEO and TCGA databases were significantly different (Figs. 2D-E). After that, qRT-PCR results from 40 patient samples were used to calculate patient risk scores according to the respective gene regression coefficients. The median was taken as CUTOFF, and the corresponding prognoses of different risk groups reported significant differences (Fig. 2F). Both univariate and multivariate analyses confirmed that risk score could be used as a prognostic factor (Figs. 2G-H). The plotted ROC curve indicated that the risk score of each factor was 0.828, which validated the reliability of risk scores. In terms of prognosis time, the reliability of prediction at one year was the highest but then gradually decreased with time (Figs. 2I-J).

### Function verification of prognosis-related genes

**Figure 3:**
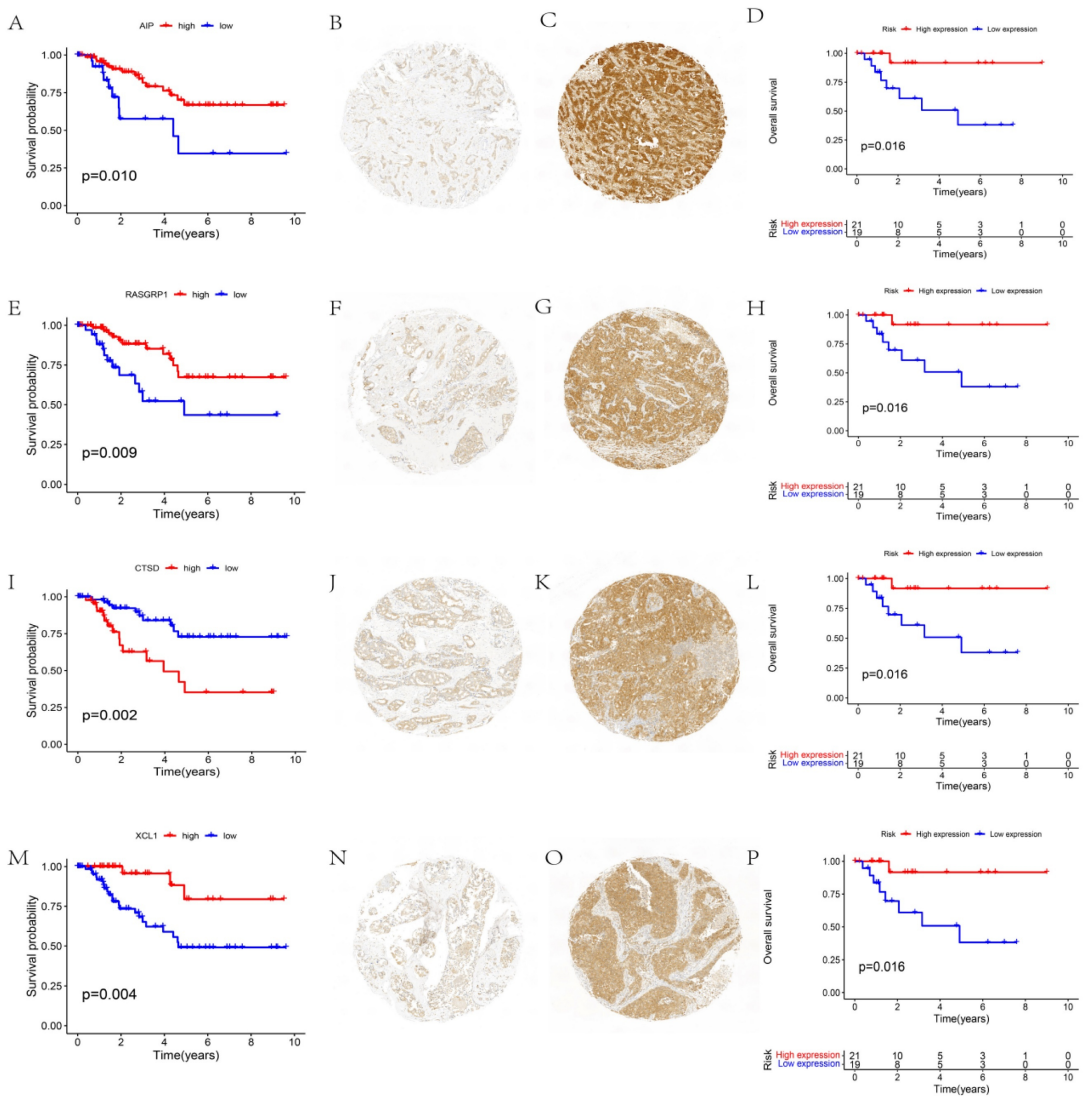
** Function validation of AIP, RASGRP1, CTSD, and XCL1. (A)** Survival analysis based on AIP expression in TCGA. **(B)** Conditions for low expression of AIP. **(C)** Conditions for high expression of AIP. **(D)** Survival analysis based on immunohistochemistry. **(E)** Survival analysis based on RASGRP1 expression in TCGA. **(F)** Conditions for low expression of RASGRP1. **(G)** Conditions for high expression of RASGRP1. **(H)** Survival analysis based on immunohistochemistry. **(I)** Survival analysis based on CTSD expression in TCGA. **(J)** Conditions for low expression of CTSD. **(K)** Conditions for high expression of CTSD. **(L)** Survival analysis based on immunohistochemistry. **(M)** Survival analysis based on XCL1 expression in TCGA. **(N)** Conditions for low expression of XCL1. **(O)** Conditions for high expression of XCL1. **(P)** Survival analysis based on immunohistochemistry.

The function of independent prognostic genes AIP, RASGRP1, CTSD, and XCL1 were further validated. The prognoses of different gene expression groups (i.e., from TCGA) were first analyzed. Subsequently, the gene expression groups were distinguished by immunohistochemistry, and the prognostic conditions of different expression groups were analyzed. These results reported that groups with different expressions of AIP (Figs. 3A-D), RASGRP1 (Figs. 3E-H), CTSD (Figs. 3I-L), and XCL1 (Figs. 3M-P) represented significantly different prognoses. High expressions of AIP, RASGRP1, and XCL1 corresponded to a better prognosis, while high expression of CTSD corresponded to a poorer prognosis.

### Understanding the possible mechanisms of CD8^+^ T cell status change

**Figure 4:**
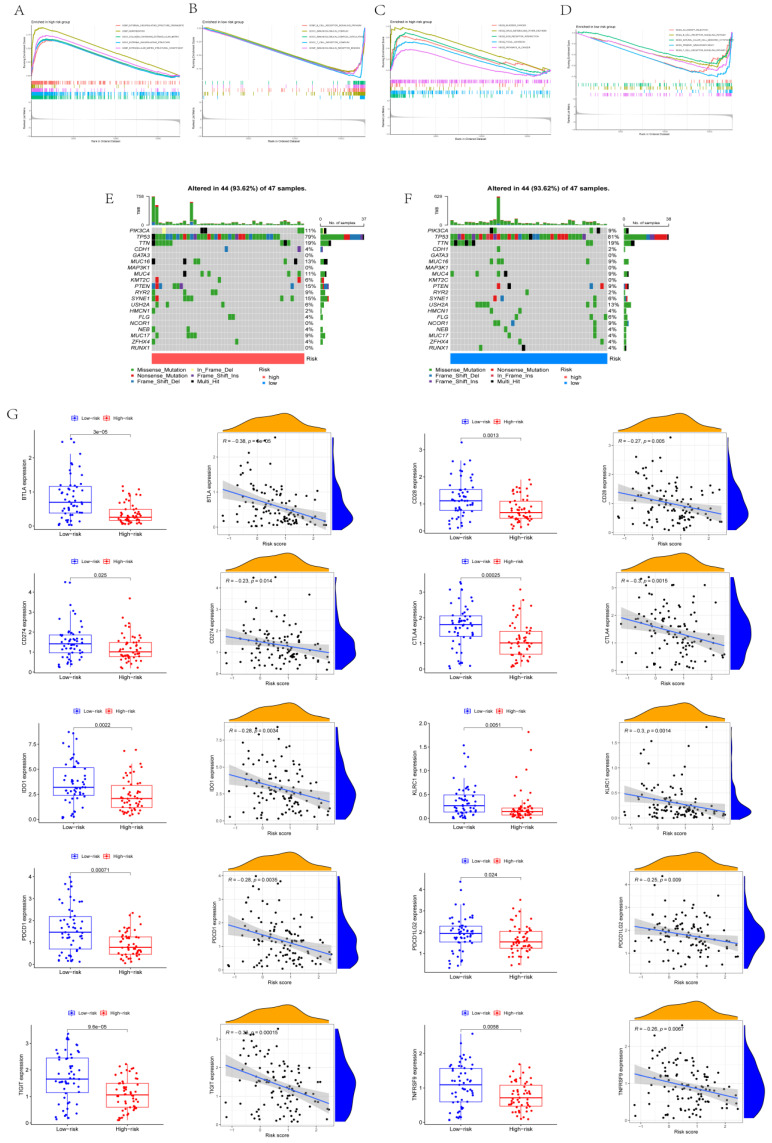
** Mechanisms of CD8^+^ T cell status change. (A-B)** Results of GO analysis. **(C-D)** Results of KEGG analysis. **(E-F)** Waterfall map. **(G)** Expression of immune checkpoints in different risk scores and correlation analysis.

The results of GO analysis and KEGG analysis using GSEA are displayed in Figs. 4A-D. Based on GO analysis, the high-risk group was mainly enriched in an external encapsulating structure organization, keratinization, collagen-containing extracellular matrix, external encapsulating structure, and extracellular matrix structural constituents. Based on GO analysis, the low-risk group was mainly enriched in B cell receptor signaling pathways, immunoglobulin complex, immunoglobulin complex circulation, T cell receptor complex, and immunoglobulin receptor binding. Based on KEGG analysis, the high-risk group was mainly enriched in bladder cancer, drug metabolism, ECM receptor interaction, focal adhesion, and cancer pathways. Based on KEGG analysis, the low-risk group was mainly enriched in allograft rejection, B cell receptor signaling pathways, natural killer cell-mediated cytotoxicity, primary immunodeficiency, and T cell receptor signaling pathways*.* From the waterfall map combined with tumor mutation load, TP53 tumor mutation was considered the most obvious (Figs. 4E-F). The immunity of different risk groups was evaluated for their correlation with risk scores. The genes BTLA, CD28, CD274, CTLA4, IDO1, KLRC1, PDCD1, PDCD1LG2, TIGIT, and TNFRSF9 reported significant differences in the different risk groups, and they were significantly correlated with risk scores (Fig. 4G).

### Correlation between risk score and immunotherapy

**Figure 5:**
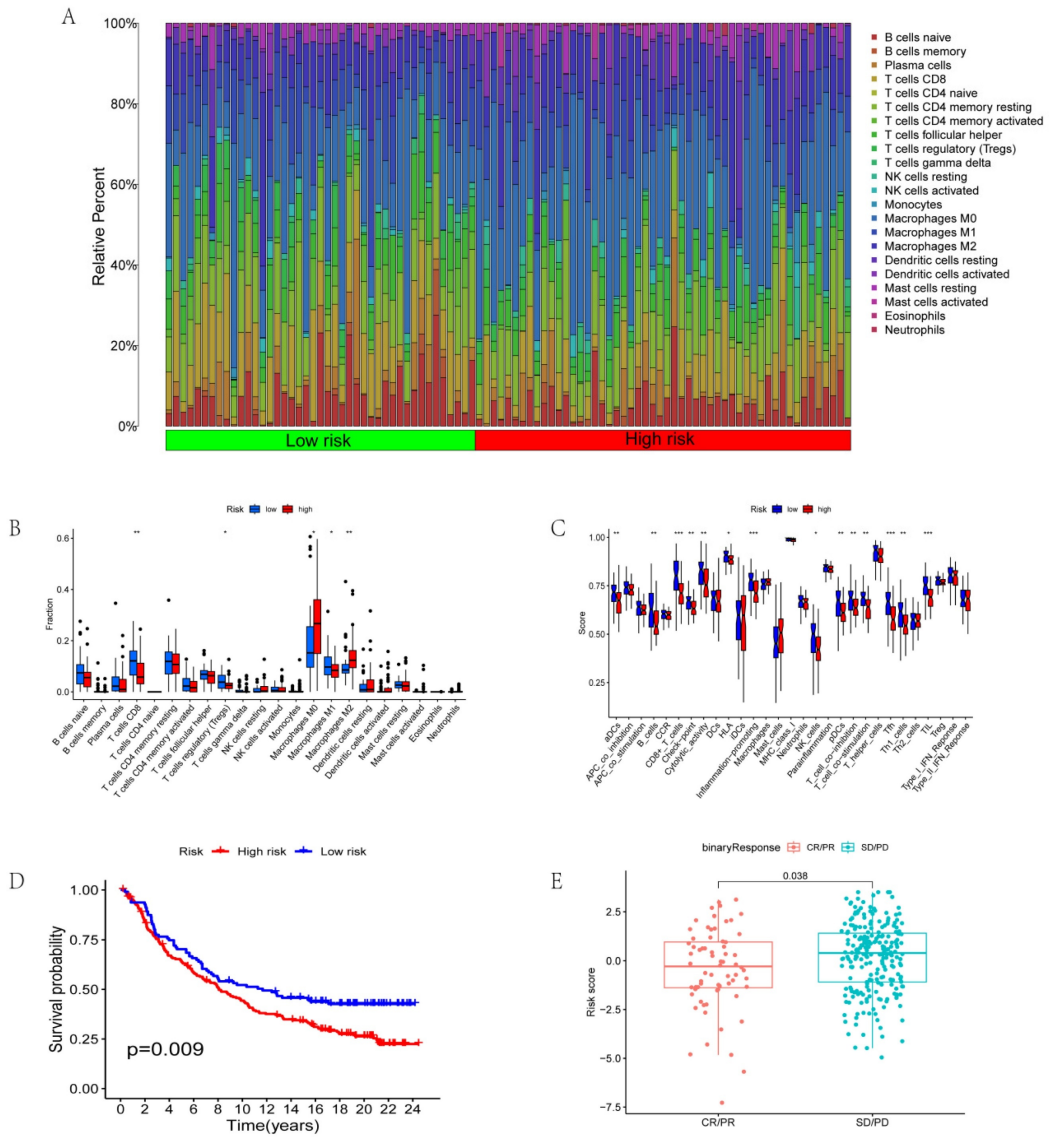
** Correlation between risk score and immunotherapy. (A-B)** Invasion of immune cells in different risk groups. **(C)** Immune function in different risk groups. **(D)** Survival analysis based on risk scores of the jarrydmartinx/metabric2 database. **(E)** Correlation between risk score and efficacy of immunotherapy.

We further analyzed immune cell invasion in patient samples and identified the immune cells with significant differences in the different groups. The immune cells included CD8^+^ T cells, T regulatory cells (Tregs), macrophage M0, macrophage M1, and macrophage M2 (Figs. 5A-B). Immune functions with significant differences in the different risk scores include aDCs, immune checkpoints, inflammation-promoting functions, pDCs, and T cell co-inhibition (Fig. 5C). The data from the jarrydmartinx/metabric2 database further verified the different prognosis of immunotherapy patients and immune scores. The results revealed that the prognosis of patients in the low-risk group was significantly better than that in the high-risk group after immunotherapy (Fig. 5D). Likewise, the efficacy of immunotherapy in the low-risk group was significantly better than that in the high-risk group (Fig. 5E).

## Discussion

This study focused on the single-cell sequencing results of CD8^+^ T cells. From the pool of differentially expressed genes, AIP, CTSD, RASGRP1, and XCL1 were selected as core genes, which could be used as independent prognostic factors. This finding was validated by immunohistochemistry and other prognostic analyses. The Lasso regression model was constructed based on the core genes, and the reliability of the prognosis prediction function of the model was verified by TCAG data and GEO data. Results from qRT-PCR further verified the accuracy of the model for prognosis prediction. We also identified differences in immune cell infiltration and function among different risk scores, and we found that the risk score model could be used to predict immunotherapy response and the prognosis of patients post-immunotherapy.

The altered response of different T cell states to tumors is multifactorial, and the exact mechanism remains unclear. The study also reported that AIP, CTSD, RASGRP1, and XCL1 were significantly differentially expressed in cells of different states and could be independent prognostic factors. AIP (aryl hydrocarbon receptor interacting protein), also known as XAP2, was initially identified as a negative regulator of hepatitis B virus X proteins, but recent studies have revealed that AIP could also regulate estrogen signaling via estrogen receptors 25, 26. AIP interacts with the CARMA1-BCL10-MALT1 complex in T cells to enhance IKK/NF-κB signaling and T cell activation, and we believe it is also a possible mechanism by which AIP regulates the function of CD8+ T cells 27. In contrast, CTSD (cathepsin D) is involved in cell apoptosis 28. Recent studies have reported that CTSD enhances the invasion and metastasis of breast cancer by promoting liver proteinase-ubiquitin-proteasome degradation 29. Additionally, CTSD could be used as a relevant target and biomarker for antibody-based therapy in TNBC patients 30. CTSD affects MDSC (Myeloid-derived suppressor cells) death by interrupted autophagy and ER stress, while MDSCs inhibit cytotoxic T lymphocytes (CTLs) and NK cell functions to promote tumor immune escape and progression. We speculate that interrupted autophagy and ER stress are the possible mechanisms by which CTSD affects the function and activity of CD8+T cells in triple negative breast cancer 31. RASGRP1 (RAS guanyl releasing protein 1), a bi-functional regulator that promotes acute inflammation and inhibits inflammation-associated cancers, can inhibit the growth of inflammation-associated tumors and activate inflammatory response by sponging let-7a to promote IL-6 expression 32. RASGRP1 is a unique biomarker for colorectal cancer and is located in the EGFR pathway 33. Its mutations are linked to immunodeficiency, immune dysregulation, and EBV-induced lymphoma 34. However, studies related to breast cancer are lacking. XCL1 (X-C motif chemokine ligand 1) is a C motif chemokine ligand, mainly produced by activated CD8^+^ T cells and natural killer cells, and it is involved in activating immune checkpoint regulators in tumors 35.

We hypothesize that Rasgrp1 influences its function and immunotherapy effect by regulating Ras-MAPK and Ras/ERK pathway in CD8+T cells. RASGRP1 also play key role at the crossroad of pathways required for the expansion of activated T lymphocytes by regulating CTPS1, which is the potential mechanism by which RASGRP1 affects functions of CD8+T cells 36-39. Hence, XLC1 is associated with tumor cell proliferation, invasion, angiogenesis, chemotherapy resistance, and other prognoses 40. Moreover, some studies have confirmed that XCL1/Glypican-3 fusion genes can induce anti-tumor cell immunity and enhance the efficacy of anti-PD-1 41. XCL1 enhances antitumor responses of CD8+ T cell through recruitment of CXCL9-expressing conventional type-1 dendritic cells and activating notch pathway, which is the potential mechanism by which XCL1 affects functions of CD8+T cells in TNBC 42.

The predictive model in this study was constructed based on public data and was further validated with real patient samples. Our study also reported that risk score correlated with immune cell infiltration and immune function changes in patients. We observed significant differences in risk scores among BTLA, CD28, CD274, CTLA4, and other immune checkpoints, indicating that changes in T cell status may affect the immune microenvironment and efficacy of immunotherapy. Subsequent findings confirmed that different risk scores resulted in significant differences in immunotherapy response and also affected the prognosis of immunotherapy patients. Therefore, our risk model could potentially predict the effect of immunotherapy in patients and identify potential gene targets to enhance the efficacy of immunotherapy.

The mechanism of change in CD8^+^ T cell status remains unclear. In this study, the differential expressions of AIP, RASGRP1, CTSD, and XCL1 were reported to induce changes in CD8^+^ T cell status. This study also identified several downstream enrichments of the differentially expressed genes (i.e., AIP, RASGRP1, CTSD, and XCL1). In addition, there are certain limitations in the study, including the reliance on retrospective data, the potential impact of tumor heterogeneity on gene expression and immunotherapy response, lack of further studies of TNBC subtypes, and the lack of follow-up validation of large amounts of clinical data. Therefore, future research should expand on these findings.

## Figures and Tables

**Table 1:** Patient information from TCGA and GSE47994 (n=249 patients)

Variables		No. Patients (%)
**Gender**		
	Female	249 (100.0%)
	Male	0 (0.0%)
**Age (y)**		
	≤ 55	147 (59.0%)
	>55	102 (41.0%)
**Tumor size**		
	T1	53 (21.3%)
	T2	168 (67.5%)
	T3	18 (7.2%)
	T4	10 (4.0%)
**Nodal status**		
	N0	113 (45.4%)
	N1	74 (29.7%)
	N2	43 (17.3%)
	N3	19 (7.6%)
**Metastasis**		
	M0	132 (53.0%)
	M1	102 (41.0%)
	Mx	15 (6.0%)
**Pathological grade**		
	G1	35 (14.1%)
	G2	73 (29.3%)
	G3	141 (56.6%)
**Stage**		
	I	50 (20.1%)
	II	79 (31.6%)
	III	68 (27.3%)
	IV	102 (41.0%)
**Ki-67**		
	< 15%	43 (17.3%)
	≥ 15%	206 (82.7%)

**Table 2:** Patient information from the Sun Yat-sen University Cancer Center (n=40 patients).

Variables		No. Patients (%)
**Gender**		
	Female	40 (100.0%)
	Male	0 (0.0%)
**Age (y)**		
	≤ 55	29 (72.5%)
	>55	11 (27.5%)
**Tumor size**		
	T1	3 (7.5%)
	T2	28 (70.0%)
	T3	5 (12.5%)
	T4	4 (10.0%)
**Nodal status**		
	N0	16 (40.0%)
	N1	10 (25.0%)
	N2	9 (22.5%)
	N3	5 (12.5%)
**Metastasis**		
	M0	36 (90.0%)
	M1	2 (5.0%)
	Mx	2 (5.0%)
**Pathological grade**		
	G1	6 (15.0%)
	G2	12 (30.0%)
	G3	22 (55.0%)
**Stage**		
	I	3 (7.5%)
	II	16 (40.0%)
	III	19 (47.5%)
	IV	2 (5.0%)
**Ki-67**		
	< 15%	11 (27.5%)
	≥ 15%	29 (72.5%)
